# Clonal dispersal is associated with tumor heterogeneity and poor prognosis in colorectal cancer

**DOI:** 10.1016/j.isci.2025.112403

**Published:** 2025-04-10

**Authors:** Selami Baglamis, Vivek M. Sheraton, Sanne M. van Neerven, Adrian Logiantara, Lisanne E. Nijman, Laura A. Hageman, Nicolas Léveillé, Clara C. Elbers, Maarten F. Bijlsma, Louis Vermeulen, Przemek M. Krawczyk, Kristiaan J. Lenos

**Affiliations:** 1 Amsterdam UMC, University of Amsterdam, Laboratory for Experimental Oncology and Radiobiology, 1081 BT Amsterdam, the Netherlands; 2 Cancer Center Amsterdam, Amsterdam, the Netherlands; 3 Oncode Institute, Amsterdam, 3521 AL Utrecht, the Netherlands; 4 Amsterdam UMC, University of Amsterdam, Amsterdam Gastroenterology Endocrinology Metabolism, Meibergdreef 9, Amsterdam, the Netherlands; 5 University of Amsterdam, Informatics Institute, Computational Science Lab, 1090 GH Amsterdam, the Netherlands; 6 University of Cambridge, Wellcome Trust–Cancer Research UK Gurdon Institute, Cambridge CB2 1QN, UK; 7 Genentech, Department of Discovery Oncology, South San Francisco, CA 94080, USA; 8 Amsterdam UMC, University of Amsterdam, Department of Medical Biology, 1105 AZ Amsterdam, the Netherlands

**Keywords:** Cell biology, Bioinformatics, Cancer

## Abstract

Clonal dispersal, resulting from the intermingling of tumor cell subpopulations, is thought to be a key driver of tumor heterogeneity. Despite advances in spatial modeling of cancer biology, quantification of clonal dispersal has been challenging. This study introduces a straightforward method, relying on fluorescent cell barcoding, to quantify clonal dispersal in various *in vitro* and *in vivo* models of colorectal cancer (CRC). Our approach allows for precise localization of clones and uncovering the degree of clonal mixing across different CRC models. Our findings suggest that clonal dispersal is correlated with the expression of genes involved in epithelial-mesenchymal transition and CMS4-related signaling pathways. We further identify a dispersal gene signature, associated with intratumor heterogeneity, which is a robust clinical predictor of poor prognosis and recurrence in CRC, highlighting its potential as a prognostic marker and a putative direction for therapeutic targeting.

## Highlights

• Clonal dispersal is quantifiable and cell line specific
• Dispersal is associated with EMT and the mesenchymal CMS4 CRC subtype
• Dispersal correlates with intratumor heterogeneity and poor clinical outcome

## Graphical abstract

**Figure:**
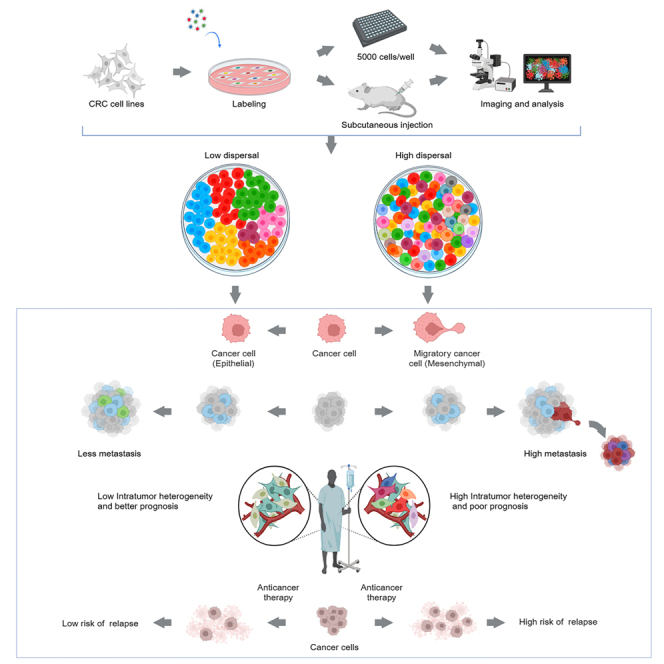

## Introduction

Colorectal cancer (CRC) is among the most common and deadly cancers worldwide making it a leading cause of cancer-related deaths.^1^^,^^2^ The course of the disease is influenced by high levels of heterogeneity, as this is associated with poor disease outcome and resistance to therapies.^3^^,^^4^ Although much emphasis has been put on genomic and epigenetic features of cancer heterogeneity and evolution, the contributions of the spatial organization of different clonal subpopulations within a tumor have been much less studied.^5^^,^^6^^,^^7^

In cancer biology, dispersal refers to the movement of cancer cells from their origin in the primary tumor to other locations within the tumor and distant metastatic sites where they resume proliferation.^8^^,^^9^^,^^10^ While clonal dispersal is frequently examined in relation to metastasis, its role in tumor evolution has been studied less vigorously. This gap limits our understanding of cancer cells migration and colonization, which might influence tumor heterogeneity and progression beyond the metastatic stage. As we demonstrated previously, clonogenic potential of cancer cells is spatially defined and restricted by external factors,^11^^,^^12^ which is also limiting genetic heterogeneity. Clonal dispersal arises from the intermingling of genetically defined tumor cell populations and could significantly impact intratumor genetic heterogeneity, thereby affecting key tumor-related processes, including therapy response, invasive (re)growth, or the development of chemotherapy resistance.^12^^,^^13^^,^^14^^,^^15^^,^^16^ During migration, cancer cells become more invasive and begin to move independently, undergoing changes in morphology and often adopting elongated shapes that are characteristic of the epithelial-mesenchymal transition (EMT).^10^^,^^17^^,^^18^ Importantly, Waclaw et al. demonstrated that higher dispersal rates elevate the probability of tumor regrowth, especially in more aggressive cancers. Moreover, if resistant mutations further enhance dispersal, regrowth is anticipated to occur even more rapidly.^19^ Given these findings, inhibition of dispersal could potentially delay the onset of recurrence and improve patient survival.^20^^,^^21^ Indeed, Shannon et al. showed that blocking the mitogen-activated protein kinase/extracellular signal regulated kinase (MAPK/ERK) pathway with the mitogen-activated extracellular kinase (MEK) inhibitor PD0325901 effectively impedes cancer cell dispersal.^20^ Another study found that activating α5β1 integrin with dexamethasone promotes the assembly of fibronectin into a dense matrix, increasing tumor cohesion by essentially “gluing” the cells together, and prevents cells from detaching from the tumor mass, reducing both their dispersal and invasion into the surrounding normal tissue.^21^

Recent advances in multiplex methods, such as protein barcoding, CRISPR barcoding, and lineage tracing combined with high-throughput sequencing, have provided valuable insights into clonal dynamics and spatial organization.^22^^,^^23^^,^^24^^,^^25^ While the current understanding of the links between the genomic and epigenetic features of cancer heterogeneity and evolution is substantial, much less is known about the contribution of spatial organization of different clonal subpopulations within a tumor.^5^^,^^6^^,^^7^ To address this knowledge gap, here we describe a simple methodology for quantifying clonal dispersal and apply it to various *in vitro* and *in vivo* CRC models. Using this method, we identify a signature of 5 genes, strongly correlating with high dispersal in our experiments, that is able to predict poor prognosis—as determined by a number of metrics—in clinical datasets.

## Results

### Generation and characterization of fluorescently barcoded CRC cell lines

**Figure 1:**
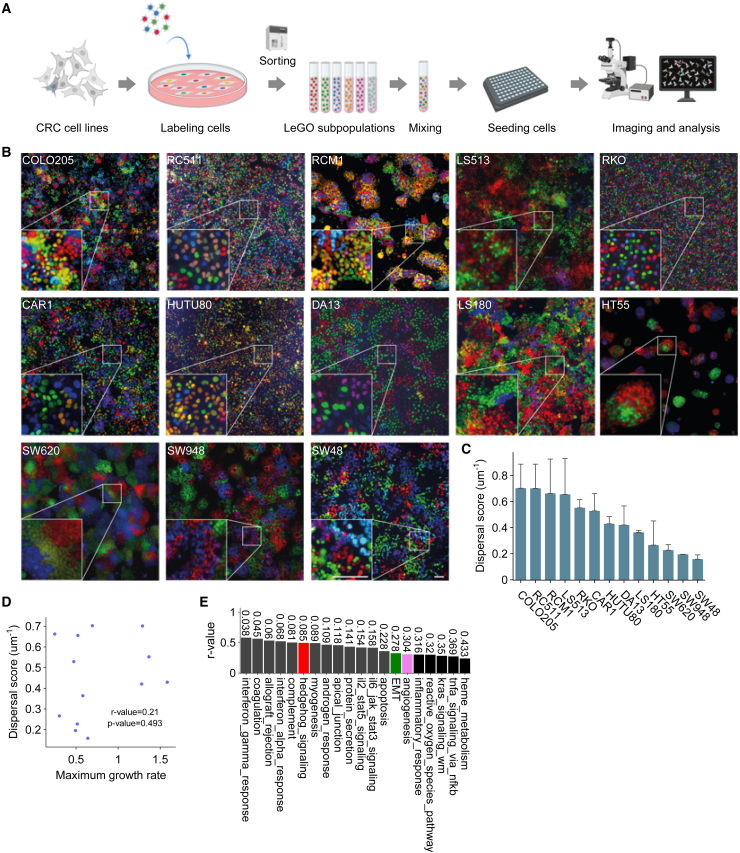
High clonal dispersal correlates with increased activity of key processes and signaling pathways *in vitro* (A) Schematic overview of the experimental pipeline for quantifying clonal dispersal *in vitro*. Created with BioRender.com. (B) Representative images of the indicated cell lines (*n* = 3), transduced with LeGO-NLS constructs. Scale bar: 100 μm, applies to all images. (C) Dispersal scores of indicated cell lines, mean + SD. (D) Pearson’s correlation between the dispersal score and growth rate. (E) Correlation between the dispersal score in CRC cell lines (*n* = 13) and expression of human MSigDB hallmark gene sets (Broad Institute).^34^ The numbers on the bars indicate the *p* values. Significance was assessed using unpaired Student’s t tests.

Quantifying clonal behavior requires the ability to distinguish between different clones (i.e., cell progenies). Fluorescent cell barcoding is an approach that achieves this goal by labeling cell populations with fluorescent tags of different colors, allowing for monitoring of multiple subpopulations under various conditions using low-resolution fluorescence microscopy.^12^^,^^26^^,^^27^^,^^28^ To track and quantify clonal behavior, we used a modified version of the lentiviral gene ontology (LeGO) optical tags,^26^^,^^28^^,^^29^ fused to the human nuclear localization signal (NLS) (Figure S1A),^30^ which enables detection of individual cell nuclei, enhancing the precision of cell localization and quantification (Figure S1B).^31^^,^^32^^,^^33^ A set of 13 CRC cell lines was transduced with these LeGO-NLS vectors (Figures 1A and 1B), and each sorted into six isogenic populations defined by stable expression of distinct color combinations: red, green, blue, orange (red + green), purple (red + blue), and cyan (green + blue) (Figures S1C–S1F). These populations were then mixed in equal ratios and used in all subsequent experiments to facilitate the detection and identification of individual clones based on the presence or absence of each tag, rather than on the intensity of each tag (Figure 1B).

### High clonal dispersal correlates with increased activity of key processes and signaling pathways *in vitro*

To study clonal behavior, the LeGO-NLS cells were seeded into 96-well plates and imaged after reaching approximately 75% confluency, using a wide-field fluorescence microscope (Figures 1A and 1B). We observed varying degrees of clonal dispersal among the different cell lines. COLO205, RC511, RCM1, LS513, RKO, CAR1, and HUTU80 exhibited relatively high intermixing, while clones in SW620, SW948, and SW48 clustered together, leading to the formation of clearly demarcated colonies (Figure 1B). Image analysis was performed using QuPath and custom Python scripts (Figure S2 and Methods S1)^33^ to calculate a dispersal score, which reflects how cells are spatially distributed in relation to each other by considering the Euclidean distance and the ordering of nearest neighbors from different clonal populations. This way, higher dispersal scores indicate increased clonal intermingling. The dispersal score was highest in COLO205 and RC511 and lowest in SW980, SW620, and SW48 (Figure 1C). Of note, dispersal was not depended on cell proliferation rates (Figures 1D, S3A, and S3B).

To investigate the transcriptional programs and signaling pathways associated with dispersal, we correlated gene expression data of the CRC cell lines with the experimentally defined *in vitro* dispersal score. This revealed a positive correlation between the dispersal score and gene signatures reflecting several key processes and signaling pathways including EMT, angiogenesis, and Hedgehog (Figure 1E).

Combined, these results suggest that clonal dispersal is a cell line-specific trait that is not dependent on cell proliferation rates but positively correlated with several signaling pathways of key importance in cancer.

### Cell-specific dispersal scores are maintained within xenograft models *in vivo*

**Figure 2:**
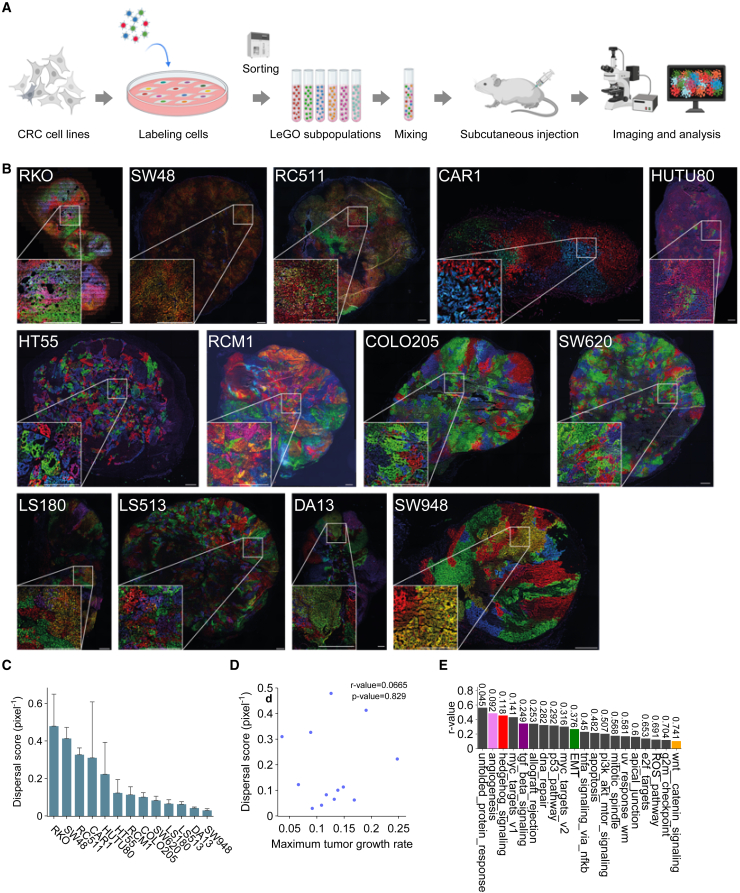
Cell-specific dispersal scores are maintained within *in vivo* xenograft models (A) Schematic overview of the experimental pipeline for quantifying clonal dispersal *in vivo*. Created with BioRender.com. (B) Representative images of xenografts formed by the indicated LeGO-NLS-transduced cell lines (*n* = 3). Scale bar: 500 μm, applies to all images. (C) Quantification of the dispersal score in xenograft sections, mean + SD. (D) Pearson’s correlation between the dispersal score and xenograft growth rate. (E) Correlation between the xenograft dispersal score and expression of human MSigDB hallmark gene sets (Broad Institute).^34^ The numbers on the bars indicate the *p* values. Significance was assessed using unpaired Student’s t tests.

To extend our analysis to a setting that includes a more relevant tumor microenvironment, we employed a subcutaneous CRC mouse model (Figure 2A) by injecting the different LeGO-NLS cell lines into both flanks of athymic nude mice. Subcutaneous tumor volume was measured in time, and growth rates for each cell line were determined (Figures S3C and S3D). Tumors were isolated at various sizes for further analysis. Similar to what we found *in vitro*, we observed a considerable variation in the clonal intermingling between the different cell lines within the xenografts (Figure 2B). We then determined the dispersal score for each xenograft and found variation among the different cell lines (Figure 2C), which, again consistent with *in vitro* findings, did not correlate with tumor growth rates (Figures 2D and S3D). With a few exceptions, most cell lines showed comparable dispersal scores *in vitro* and *in vivo* (Figure S3E).

In line with the gene set correlations observed with *in vitro* dispersal scores, we found a positive correlation between *in vivo* dispersal scores and processes including angiogenesis and EMT, as well as signaling pathways involved in EMT, such as Hedgehog, transforming growth factor β (TGF-β), and Wnt/β-catenin (Figure 2E).^35^

The dispersal score thus appears to be a simple yet useful metric for assessing the intermingling of CRC cells, recapitulating the heterogeneity in dispersal both for *in vitro* and *in vivo* cancer cell populations. Reassuringly, the dispersal score is associated with several signaling pathways involved in EMT and cancer progression in both *in vitro* and *in vivo* contexts.

### A robust dispersal gene signature is associated with EMT and the mesenchymal CRC subtype

**Figure 3:**
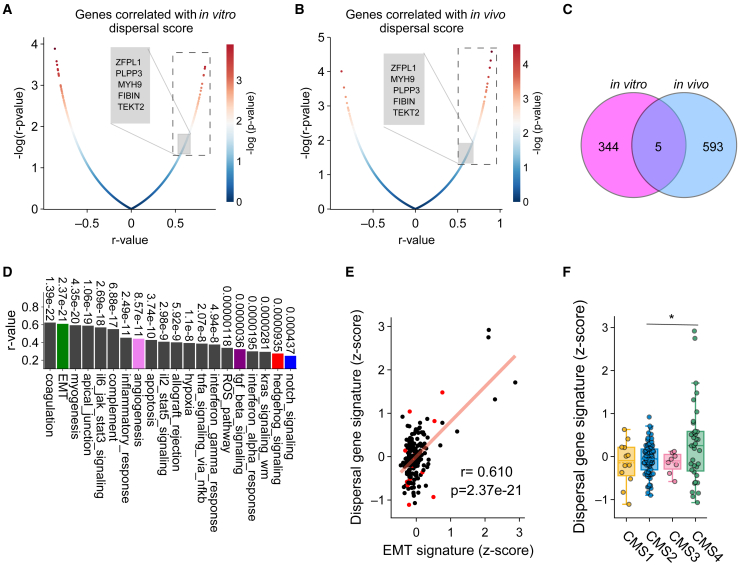
A robust dispersal gene signature based on experimentally observed clonal dispersal (A and B) Correlation between gene expression and clonal dispersal of CRC cell lines *in vitro* (A) and *in vivo* (B). Significantly positively correlated genes are indicated by the dashed boxes. (C) Venn diagram showing numbers of genes that are positively and significantly correlated with the dispersal scores in either *in vitro* or *in vivo* models. Overlapping genes (*n* = 5) were assigned to the dispersal gene signature. (D) Correlation dispersal gene signature expression in CRC lines (*n* = 196) and various pathways from the human MSigDB hallmark gene set collection (Broad Institute).^34^ (E) Correlation between the dispersal gene signature and EMT signature in 196 CRC cell lines. Each dot represents a different CRC cell line, and red dots represent the cell lines used in this study. (F) Dispersal gene signature in CMS1 (*n* = 12), CMS2 (*n* = 79), CMS3 (*n* = 8), and CMS4 (*n* = 36) CRC cell lines. Each dot represents a different CRC cell line. Significance was assessed using unpaired Student’s t tests. ∗*p* < 0.05.

To further study how clonal dispersal is associated with gene expression profiles and tumor characteristics, we attempted to construct a signature of genes that positively correlated with the experimentally defined dispersal scores both *in vitro* and *in vivo*. To this end, we correlated gene expression profiles of the 13 CRC cell lines used for the *in vitro* (Figure 1) and *in vivo* (Figure 2) dispersal determination experiments with the obtained dispersal scores in those experiments. We compared the overlap between genes that positively correlated with *in vitro* and *in vivo* dispersal scores, resulting in a set of 5 genes that strongly correlated with both scores: *MYH9*, *ZFPL1*, *TEKT2*, *PLPP3*, and *FIBIN* (Figures 3A–3C; Table S1).

These genes have been reported to be involved in several cancer-related processes; i.e., MYH9 (Myosin Heavy Chain 9) is involved in diverse biological processes such as cell adhesion, polarity, migration, division, and signal transduction. It has been identified as a tumor promoter in various cancers and contributes to cell proliferation, EMT, invasion, metastasis, chemoradiotherapy resistance, stemness maintenance, and metabolic regulation metabolism.^36^^,^^37^ ZFPL1 (Zinc Finger Protein Like 1) has been suggested to regulate the PI3K-Akt survival pathway, thereby governing processes like tumor growth, migration, proliferation, and metabolism. This pathway is recognized as a core oncogenic signaling mechanism.^38^^,^^39^^,^^40^ TEKT2 (Tektin2) is a microtubule-associated protein, which plays a crucial role in regulating sperm flagellum movement and has been identified as a prognostic marker in cervical cancer patients.^41^^,^^42^ PLPP3 (Phospholipid Phosphatase 3) promotes the β-catenin/lymphoid enhancer-binding factor 1 (β-catenin/LEF-1) axis, facilitating endothelial cell migration, cell-cell adhesion, and branching structure formation.^43^ Lastly, FIBIN (Fin Bud Initiation Factor Homolog) has been associated with key signaling pathways, including extracellular matrix interactions, PI3K-Akt signaling, and extracellular matrix-receptor interactions.^44^

Using this cross-model “dispersal signature” on an extended dataset of 196 RNA sequencing samples of CRC models, including cell lines, primary cell lines, and spheroids, we again observed a strong correlation between the expression of the dispersal signature genes and the genes involved in EMT and angiogenesis, as well as TGF-β, Hedgehog, and Notch signaling (Figures 3D and 3E). Both EMT and TGF-β signaling are strongly associated with invasion and metastasis in CRC and are known features of the poor-prognosis CMS4 subtype.^45^^,^^46^ Indeed, comparing cell lines that were classified as CMS2 (the canonical CRC subtype) with CMS4 cell lines, we observed an increased expression of the dispersal gene set in the CMS4 group (Figure 3F).

### Dispersal signature is associated with negative clinical outcomes

**Figure 4:**
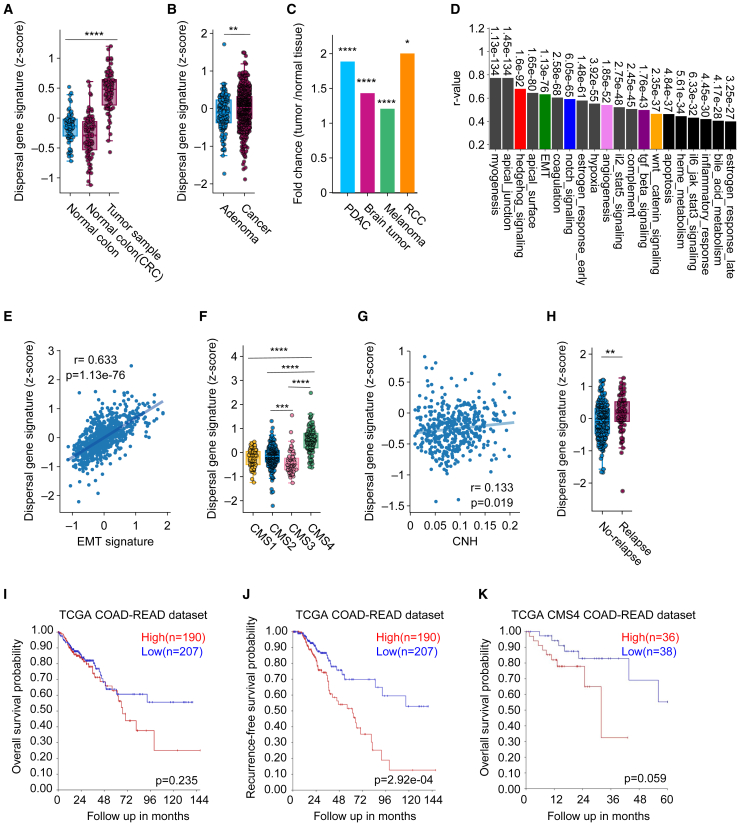
Dispersal signature correlates with negative clinical outcomes (A) Dispersal gene signature (*Z* score) in colon tissue of healthy controls (*n* = 72), normal colon tissue of CRC patients (*n* = 77), or CRC tissue (*n* = 132) (GSE199057 dataset).^47^ (B) Dispersal gene signature (*Z* score) in CRC adenoma (*n* = 132) and cancer patients (*n* = 573) in a microarray meta-dataset.^48^^,^^49^^,^^50^^,^^51^^,^^52^^,^^53^^,^^54^^,^^55^^,^^56^^,^^57^^,^^58^^,^^59^ (C) Fold change of dispersal signature gene expression between normal and tumor tissue, in different cancer types. (D) Correlation between dispersal gene signature expression and human MSigDB hallmark gene sets in CRC samples (*n* = 673, TCGA COAD-READ dataset).^60^ (E) Correlation between dispersal gene signature and EMT signature in CRC samples (*n* = 673, TCGA COAD-READ dataset).^60^ Each dot represents a different tumor sample. (F) Dispersal gene signature expression (*Z* score) in CMS1 (*n* = 68), CMS2 (*n* = 207), CMS3 (*n* = 64), and CMS4 (*n* = 118) CRCs (TCGA COAD-READ dataset).^60^ Each dot represents a different tumor sample. (G) Correlation between copy-number heterogeneity (CNH)^13^ and dispersal gene signature (*Z* score) in microsatellite stable (MSS) CRC tumors (TCGA COAD-READ dataset).^60^ (H) Dispersal gene signature (*Z* score) of non-relapsing (*n* = 222) and relapsed CRC tumors (*n* = 77) (GSE14333 dataset).^61^ (I and J) (I) Overall survival probability and (J) recurrence-free survival probability of CRC patients with either high or low expression of the dispersal gene signature (TCGA COAD-READ dataset).^60^ (K) Overall survival probability of CMS4 patients with either high or low dispersal gene signature (TCGA COAD-READ dataset).^60^ Significance was assessed using unpaired Student’s t tests for comparisons between two groups, ANOVA followed by a *post hoc* test for multiple group comparisons and the chi-squared test for survival analysis. Ns, not significant, ∗*p* < 0.05, ∗∗*p* < 0.01, ∗∗∗*p* < 0.001, and ∗∗∗∗*p* < 0.0001.

To investigate the relationship between the level of clonal dispersal and clinical CRC outcomes, we first examined the expression of the dispersal gene signature in various CRC patient datasets. Interestingly, the dispersal gene set was generally elevated in cancerous tissues as compared to the normal colon, and carcinoma tissue displayed increased expression compared to adenomas (Figures 4A and 4B).^47^^,^^48^ We observed similar trends in tissues from pancreatic ductal adenocarcinoma (PDAC), brain tumors, melanoma, and renal cell carcinoma (Figures 4C and S4A–S4D).

Next, we applied the dispersal gene signature on tumor samples of The Cancer Genome Atlas (TCGA) Colon-Rectum Adenocarcinoma (COAD-READ) dataset,^60^ and also here we found significant correlations with EMT, angiogenesis, and signaling pathways such as TGF-β, Hedgehog, and Notch (Figures 4D and 4E). In line with our findings in cell lines, CMS4 samples exhibited a significantly higher dispersal gene signature expression compared to the other subtypes (Figure 4F).

Since clonal dispersal could result in higher levels of intratumor heterogeneity due to increased outgrowth of clonal populations, we investigated the relation between clonal dispersal and level of intratumor heterogeneity, using previously defined copy-number heterogeneity (CNH) data obtained from CRC patients.^13^ It has been shown that chromosomal copy-number variations exhibit various levels of heterogeneity within tumors in various cancer types,^62^^,^^63^^,^^64^^,^^65^ and these levels have been associated with patient outcomes.^64^^,^^66^ Indeed, a notable correlation between expression of the dispersal gene signature and CNH within CRCs was observed (Figure 4G). Especially, CRCs with the highest CNH levels exhibited a significantly increased dispersal signature score compared to those with low or intermediate CNH (Figure S4E).

We then investigated the potential prognostic value of the dispersal signature by correlating it to various clinical outcomes. Notably, expression of signature genes did not depend on the disease progression stage (Figure S4F) but was increased in tumor samples of CRC patients that suffered from relapse of the disease (Figure 4H).^61^ Importantly, patients that have tumors with high expression of the dispersal signature also had the lowest overall, recurrence-free, and relapse-free survival probabilities (Figures 4I, 4J, S4G, and S4H).

To determine whether dispersal genes provide an advantage over the EMT signature (Table S2), which is strongly associated with CMS4, in predicting survival, we compared both signatures across CRC subtypes. As expected, EMT signature expression was elevated in all CMS4 CRCs compared to other subtypes, whereas the dispersal signature displayed a more heterogeneous expression among these samples (Figure 3F and S4I). Interestingly, while EMT signature expression effectively distinguishes good- from poor-prognosis CRCs (Figures S4J and S4K), it did not further stratify prognosis within the CMS4 subgroup (Figure S4L). In contrast, the dispersal signature successfully separated CMS4 cases into subgroups with poor and relatively better prognoses (Figure 4K).

This observation aligns with a recent study that subdivided the CMS4 subgroup into poor- and favorable-outcome groups based on transcriptomic profiles.^67^ Notably, when we plotted the dispersal gene signature across the different CRC prognostic subtypes (CRPSs) identified in this study, the poor-prognosis CMS4 samples (CRPS4) exhibited significantly higher dispersal gene expression (Figure S4M). These findings suggest that, while the EMT signature is effective in identifying CMS4 tumors, the dispersal signature provides additional value in refining prognosis within this high-risk subgroup.

In conclusion, the dispersal signature is a robust indicator of poor prognosis in CRC, beyond CMS classification, and may potentially be used as a prognostic tool to identify patients at high risk of recurrence.

## Discussion

This study provides a comprehensive analysis of clonal dispersal in CRC using fluorescent cell barcoding. Our experiments reveal that clonal dispersal is highly cell line specific and independent of cell proliferation rates, underscoring the complexity of tumor cell behavior and the potential influence of genetic and epigenetic factors.

Our results demonstrate a positive correlation between dispersal scores and gene expression associated with angiogenesis, EMT, and Hedgehog signaling, as well as with the CMS4 CRC subtype. These pathways are well known to play critical roles in cancer progression, metastasis, and tumor microenvironment modulation.^46^^,^^68^^,^^69^ Their association with high dispersal scores suggests that clonal dispersal may facilitate cancer cell adaptability and invasiveness, contributing to tumor aggressiveness.

Extending our analysis to murine xenograft models revealed a similar pattern of clonal dispersal as observed *in vitro*. Further, the dispersal scores *in vivo* correlated with the same key signaling pathways identified *in vitro*, suggesting a consistent mechanism driving dispersal across different environments. However, the lack of a statistically significant correlation between *in vitro* and *in vivo* dispersal scores for some cell lines indicates the influence of the tumor microenvironment or other factors on clonal behavior, highlighting the well-known impact of both intrinsic genetic factors and extrinsic environmental factors when studying cancer cell dynamics.^11^^,^^12^

The identification of specific genes, such as *MYH9*, *ZFPL1*, *TEKT2*, *PLPP3*, and *FIBIN*, as part of a robust dispersal gene signature further emphasizes the transcriptional underpinnings of clonal dispersal. This signature might provide a molecular framework for a better understanding of the link between clonal behavior and cancer biology, offering potential targets for therapeutic intervention.

The expression of the signature genes was elevated in cancerous tissues compared to normal colon tissues and was more pronounced in the aggressive CMS4 subtype. Interestingly, we found that CRCs with high levels of intratumor heterogeneity, as measured by CNH, likewise displayed increased expression of the dispersal gene signature, suggesting a relation between the clonal composition of a tumor and the ability of cancer cells to migrate through the tumor. We previously demonstrated the importance of the location of a tumor cell for the clonogenicity of that cell, suggesting that clonal composition or heterogeneity is restricted by the spatial distribution of the clones.^11^^,^^12^ Therefore, the ability of a cancer cell to migrate beyond clone boundaries could increase the chance of survival of individual clones, thereby increasing the genetic heterogeneity of the tumor. Intratumor heterogeneity has been associated with poor patient survival, and tumor evolution can directly impact therapy response and resistance development.^13^

In our analysis, both the dispersal and EMT gene signatures demonstrated comparable prognostic value overall, with the EMT signature slightly outperforming for overall survival, while the dispersal signature provided a stronger separation for recurrence-free survival. Notably, EMT signature effectively identified CMS4/poor-prognosis tumors but could not further stratify prognosis within the CMS4 subgroup. In contrast, the dispersal signature successfully distinguished between poor and not-so-poor prognosis within CMS4 tumors, highlighting its added utility in refining prognostic insights for this high-risk subgroup.^67^ These findings suggest that the dispersal and EMT signatures offer complementary value and could be jointly leveraged for enhanced stratification in CRC. The correlation with poor survival probabilities further emphasizes the need to explore dispersal-related pathways as potential therapeutic targets to mitigate cancer progression and improve patient outcomes.

In conclusion, our study demonstrates the utility of fluorescent cell barcoding in quantifying clonal dispersal and reveals significant correlations between dispersal behavior and key cancer signaling pathways. The identification of a dispersal gene signature provides a valuable molecular tool for predicting CRC aggressiveness and patient outcomes and establishes the relation between dispersal ability and genetic heterogeneity within a tumor. By advancing our understanding of clonal dynamics, these findings pave the way for developing targeted therapies aimed at disrupting pathways associated with high clonal dispersal, potentially improving prognosis and treatment strategies for CRC patients. Future research should continue to explore the complex interplay between clonal behavior, tumor microenvironment, and cancer progression, ultimately contributing to more effective and personalized approaches to cancer care.

### Limitations of the study

While our study provides new insights into clonal dispersal and its implications for CRC, several limitations should be acknowledged. First, the study relies on a limited number of cell lines, which may not fully capture the heterogeneity of CRC. Future studies should aim to include a broader range of cell lines and patient-derived samples to enhance the generalizability of our findings. Additionally, the mechanisms underlying the observed correlations between dispersal scores and signaling pathways are not clear and warrant further investigation to elucidate the causal relationships involved. In particular, studying clonal dispersal should be extended beyond the limited applicability of (fluorescence) barcoding, for instance by applying the emerging spatial-omics methods,^70^ which could be used for mapping clonal composition in tumors without the need for (fluorescent) tagging.

Moreover, while the dispersal signature offers a promising predictive tool, its clinical application requires validation in larger, independent cohorts. Longitudinal studies examining the evolution of dispersal signatures over time and in response to treatment will be crucial for understanding their role in cancer progression and therapy resistance.

## Resource availability

### Lead contact

Requests for further information and resources should be directed to and will be fulfilled by the lead contact, Kristiaan J. Lenos (k.j.lenos@amsterdamumc.nl).

### Materials availability

This study did not generate new unique reagents.

### Data and code availability

• This paper analyzes existing, publicly available data, accessible at FiglinQ repository (https://create.figlinq.com/∼s.baglamis/379/), R2 Genomics Analysis and Visualization Platform (https://r2.amc.nl), GEO: GSE36133, GSE100478, GSE59857, GSE68950, GSE100549, GSE100479, GSE4183, GSE8671, GSE9348, GSE15960, GSE20916, GSE21510, GSE22598, GSE23194, GSE23878, GSE32323, GSE33113, GSE37364, GSE62452, GSE4290, GSE3189, GSE53757, GSE14333, and GSE39582), MSigDB (https://doi.org/10.1016/j.cels.2015.12.004), and TCGA (https://doi.org/10.7937/K9/TCIA.2016.HJJHBOXZ). All data reported in this paper will be shared by the lead contact upon request.
• All original code has been deposited at Zenodo and is publicly available at https://doi.org/10.5281/zenodo.15125571 as of the date of publication. All other code is available in this paper’s supplemental information.
• Any additional information required to reanalyze the data reported in this paper is available from the lead contact upon request.

## Acknowledgments

S.B. is thankful for the scholarship provided by the Ministry of National Education of the Republic of Turkey. This work is supported by the Dutch Cancer Society (KWF#10529), 10.13039/501100021821Oncode Institute, 10.13039/100003194The New York Stem Cell Foundation, and grants from the 10.13039/501100000781European Research Council (ERC-CoG 101045612 - NIMICRY) and 10.13039/501100001826ZonMw (Vici 09-15018-21-10029) to L.V. L.V. is a New York Stem Cell Foundation – Robertson Investigator.

## Author contributions

Conceptualization, K.J.L., P.M.K., L.V., V.M.S., S.M.v.N., M.F.B., N.L., and C.C.E.; methodology, K.J.L., P.M.K., L.V., S.M.v.N., and S.B.; software, V.M.S.; formal analysis, S.B. and K.J.L.; investigation, S.B., K.J.L., S.M.v.N., A.L., L.E.H., and L.A.H.; data curation, V.M.S.; writing – original draft preparation, S.B.; writing – review and editing, K.J.L., P.M.K., L.V., V.M.S., S.M.v.N., and M.F.B.; visualization, S.B.; supervision, K.J.L., P.M.K., L.V., and S.M.v.N.; project administration, K.J.L., P.M.K., and L.V.; funding acquisition, L.V. and C.C.E. The final version of the manuscript was approved by all authors.

## Declaration of interests

L.V. received consultancy fees from Bayer, MSD, Servier, and Pierre Fabre, but these had no relation to the content of this publication. L.V. is presently an employee of Genentech, Inc.

## STAR★Methods

### Key resources table

**Table:** 

REAGENT or RESOURCE	SOURCE	IDENTIFIER
**Bacterial and virus strains**		

3rd generation lentiviral system	AddGene/Trono lab^71^	N/A

**Chemicals, peptides, and recombinant proteins**		

DMEM/F12	Gibco	Cat#11330-032
RPMI	Gibco	Cat#52400–025
DMEM	Gibco	Cat#11965-092
Glucose	Gibco	Cat#A24940-01
Sodium Pyruvate	Gibco	Cat#11360-070
Lipofectamine 2000	Invitrogen	Cat#11668–019

**Deposited data**		

Cell line RNAseq datasets	GEO	GSE36133,^72^GSE100478,^73^^,^^74^GSE59857,^75^^,^^76^GSE68950 (NCI, 2015), GSE100549,^73^GSE100479.^73^
Microarray meta-dataset ^48^ for adenoma and cancer samples.	GEO	GSE4183,^49^GSE8671,^50^GSE9348,^51^ GSE15960,^52^GSE20916,^53^GSE21510,^54^GSE22598,^55^GSE23194,^77^GSE23878,^56^GSE32323,^57^GSE33113,^58^GSE37364.^59^
Microarray gene-expression profiles PDAC patients	GEO	GSE62452^78^
mRNA expression data of brain tumor	GEO	GSE4290^79^
Affymetrix Hu133A microarray for Melanoma	GEO	GSE3189^80^
Gene array analysis of RCC	GEO	GSE53757^81^
Colon-Rectum Adenocarcinoma (COAD-READ) dataset	TCGA	The Cancer Genome Atlas Network^60^
Relapse-free survival probability	GEO	GSE14333,^61^GSE39582^82^
Hallmark gene set collection	MSigDB	Arthur et al. 2015^34^

**Experimental models: Cell lines**		

HEK293T	ATCC	CVCL_0063
RKO	Sanger Institute	CVCL_0504
SW48	Sanger Institute	CVCL_1724
HT55	Sanger Institute	CVCL_1294
SW948	Sanger Institute	CVCL_0632
COLO205	ATCC	CVCL_0218
RCM1	Sanger Institute	CVCL_1648
LS180	Sanger Institute	CVCL_0397
LS513	Sanger Institute	CVCL_1386
CAR1	Sanger Institute	CVCL_1116
HUTU80	Sanger Institute	CVCL_1301
DA13	University of Palermo, Italy	N/A
RC511	University of Palermo, Italy	N/A
SW620	ATCC	CVCL_0547

**Experimental models: Organisms/strains**		

Hsd: Athymic Nude-Foxn1^nu^ mice	Envigo RMS B.V.	Cat# 069; RRID: IMSR_ENV:HSD-069

**Oligonucleotides**		

human c-Myc proto-oncogene (3' GGAC GACGCTTCTCCCAGTTTAACCTG 5’)	Sigma-Aldrich	Dang & Lee^30^

**Recombinant DNA**		

pMD2.G (envelope)	AddGene/Trono lab	Cat#12259
pMDLg/pRRE (packaging)	AddGene/Trono lab	Cat#12251
pRSV-Rev (packaging)	AddGene/Trono lab	Cat#12253
LeGO-C2	AddGene/Weber et al.^26^	Cat#27339
LeGO-V2	AddGene/Weber et al.^26^	Cat#27340
LeGO-Cer2	AddGene/Weber et al.^26^	Cat#27338

**Software and algorithms**		

GraphPad Prism	GraphPad Software 10.2.0	www.graphpad.com
IncuCyte™ base analysis software	Sartorius	N/A
R2 bioinformatics platform	AMC	http://hgserver1.amc.nl
QuPath version v0.5.1	QuPath	https://qupath.github.io/
FlowJo v10	BD Biosciences	*N/A*
Python 3.12.1		https://www.python.org/

**Other**		

Thunder wide-field fluorescence microscope	Leica	DMI8
FACS cell sorter	Sony	SH800
BD LSRFortessa™ Cell Analyzer	BD Bioscience	N/A
Incucyte® S3 Live-Cell Analysis system	Sartorius	S3

### Experimental model and study participant details

#### Cell culture

RKO (male donor), SW48 (female donor), HT55 (female donor), SW948 (female donor), LS180 (female donor), CAR1 (male donor), HUTU80 (male donor), SW620 (male donor) cell lines (kindly provided by Sanger Institute, Cambridge, UK) and Da13 (female donor), RC511 (female donor) cell lines (kindly obtained by University of Palermo, Palermo, Italy) were cultured in DMEM/F12 supplemented with 10% fetal calf serum and 1% Penicillin-Streptomycin. Colo205 (male donor), LS513 (male donor), and RCM1 (female donor) cell lines (kindly provided by Sanger Institute, Cambridge, UK) were maintained in RPMI medium containing 1% Glucose, 1 mM Sodium Pyruvate, 10% fetal calf serum and 1% Penicillin-Streptomycin (Gibco). HEK293T cells (female donor, ATCC) were cultured in DMEM medium supplemented with 10% fetal calf serum and 1% Penicillin-Streptomycin. Mediums and supplements are purchased from Gibco (Waltham, MA, USA). Cell lines were incubated at 37°C and 5.0 % CO_2_. Cell lines were randomly selected from both female and male donors and no gender-based differences were observed in the results. Cell lines were authenticated through short tandem repeat profiling combined with mutation analysis and were regularly tested for mycoplasma infection.

#### *In vivo* experiments

All *in vivo* experiments conducted in this study received approval from the Animal Experimentation Committee at the Amsterdam UMC in Amsterdam (AVD11800202114947 and DEC103141) and were performed under national guidelines. Female nude mice (Hsd: Athymic Nude-Foxn1nu), aged between 6 and 12 weeks, were purchased from Envigo. Only female animals were used to prevent possible serious discomfort due to fighting in male mice and all possible consequences such as injuries and individual housing of male animals during the entire experiment that might affect tumor growth (i.e. inflammatory reactions, stress). The assignment of animals to experimental groups was done randomly, and no blinding was applied during the experiments. Animals were only excluded from analysis if no tumors appeared.

### Method details

#### Nuclear lentiviral gene ontology (LeGO-NLS) markers

Standard DNA cloning protocols were used for introducing nuclear localization signal (NLS) of human c-Myc proto-oncogene (3' GGACGACGCTTCTCCCAGTTTAACCTG 5’) into LeGO-C2 (#27339), LeGO-V2 (#27340), and LeGO-Cer2 (#27338) vectors (Addgene), encode mCherry, Venus, Cerulean fluorescent coding genes respectively, for nuclear visualization.^30^^,^^31^^,^^32^^,^^33^ Lentivirus was generated by transfecting HEK293T cells with the LeGO-NLS plasmids, along with the third generation packaging plasmids^71^ pMD2.G, pMDLg/pRRE, and pRSV-Rev, using Lipofectamine 2000 (Invitrogen, Waltham, Massachusetts). The supernatant was collected 48 and 72 hours post-transfection and subsequently filtered through a 0.45 μm filter (Millipore, Germany). Then, cell lines were selected based on CMS classification,^73^ CMS1 (RKO, SW48), CMS2 (HT55, SW948, COLO205, RCM1), CMS3 (LS180, LS513), CMS4 (CAR1, HUTU80, DA13, SW620, RC511), were transduced with lentiviral gene ontology (LeGO) markers,^26^ following established protocols outlined in prior publications by Weber et al.^29^ Briefly, 50,000 cells were seeded in a single well of a 12-well plate containing 1 mL culture medium. The plate was then placed in the incubator at 37°C with 5.0 % CO_2_ for ∼24 h to reach the confluency ∼70 %. Subsequently, 50 μL of concentrated lentivirus encapsulating LeGO-NLS DNA was added into 1 mL culture medium in the presence of 8 μg mL^−1^ polybrene (Sigma-Aldrich). This mixture was incubated overnight at 37°C with 5.0 % CO_2_. After discarding the medium, cells were washed with PBS and a new medium was added to wells.

#### Cell sorting and selection

Cell lines were sorted into 6 distinct colors: red, green, blue, orange, purple, and cyan by using an SH800 Sony cell sorter (San Jose, CA, USA). These subclones’ cultures were expanded and passed once a week. Upon passaging the stability of fluorescent tags was followed for 28 days with a Fluorescence Activated Cell Sorting (FACS) BD LSR Fortessa (BD Biosciences, Franklin Lakes, NJ, USA) machine with 450-, 530-, and 616- nm lasers (Figure S1). FlowJo (FlowJo LLC) software was used to analyze the data.

#### *In vitro* experiments

To compute clonal dispersal, we initially seeded 5000 cells in 96-well plates and captured images at approximately 75 % confluency. These images were initially annotated in Qupath (Figure S2 and Method S1),^33^ and subsequently, the data were exported to run a script for clonal dispersal calculation.

Proliferation assays were conducted to calculate the maximum growth rate for each cell line (Figures S3A and S3B). To accomplish this, we plated 5000 cells in 96-well plates (Greiner, Sensoplate glass bottom, Kremsmünster, Austria) and monitored them using an Incucyte® S3 Live-Cell Analysis system (Sartorius, Göttingen, Germany) every 4-hour time intervals until the wells reached 85-90 % confluency.

#### Xenografts studies

To establish xenografts, a mixture of 50,000 LeGO-NLS tagged colorectal cancer cells in medium was combined in a 1:1 ratio with Matrigel (Corning, Bedford, MA, USA) and injected subcutaneously into both flanks of female a-thymic nude mice. Tumor growth was monitored twice a week using a caliper, using the formula 0.5 × length × width × height.

Once the mice were sacrificed, the tumors were isolated, fixed in a 4% paraformaldehyde in PBS solution overnight at 4°C, and subsequently preserved in a 20% sucrose solution for 12 hours at 4°C. Tumor sections were then obtained from the isolated tumors. To prevent the fluorescent dye from fading, Prolong Gold (ThermoFisher Scientific, Eugene, OR, USA) was applied to the tumor sections before imaging. A Leica Thunder wide-field fluorescence microscope at 10x magnification used to scan xenografts.

#### Imaging

Cells were imaged with a Leica Thunder Wide Field Fluorescence Microscope with 10x magnification. The following imaging settings were employed: Quad Filter Block (CYR71010) with EX:375-412;483-501;562-588, DC:535;505;595 and EM:441-471;512-548;600-660 parameters for imaging Cerulean, Venus, and mCherry respectively. To enhance the quality of the *in vitro* images, a Z-stack and Thunder computational clearing mode were utilized. While adaptive focus control was utilized for scanning xenografts.

#### Dispersal Score calculation and image analysis

Dispersal Score in the study was designed to quantify the mixing of cell populations per unit distance. A well-mixed population, with a high Dispersal Score, indicates an intrinsic property of cells in subpopulation(s) to displace from their initial location. Mathematically, this can be denoted as,

$$d\left(x_{i{,P}_{k}}\right)=\min_{k\neq l}d\left(x_{i{,P}_{k}},x_{j,P_{l}}\right)$$

Where i and j are indices representing members of populations $P_{k}$ and other than $P_{k}$ ($P_{l}$) other than, respectively. d is the distance metric, it is Euclidian in case of *in vivo* quantifications and neighbour order in case of *in vitro* quantifications.

$$D={\left(\frac{1}{N}\sum_{\begin{array}{c} i\\ k=1 \end{array}}^{N}\frac{1}{n_{k}}\sum_{i=1}^{n_{k}}d\left(x_{i{,P}_{k}}\right)\right)}^{-1}$$

Where N is the total number of populations, $n_{k}$ is the total number of members of a population $P_{k}$ and D is the Dispersal Score.

*In vitro* image analysis: Images were uploaded to Qupath, and the StarDist nuclei detection plug-in was executed (Figure S2 and Methods S1). Following that, each cell line was manually categorized into six classes (red, green, blue, orange, purple, cyan) and one class for background. Then a trained model was used to infer positions of clonal populations in all images and data was exported. The exported data was analyzed using Python scripts to quantify the Dispersal Score. Dispersal Score of a cell was analyzed using two metrics, Euclidean distance-based and neighbor cell order-based Dispersal Scores. They measure the least Euclidean distance and nearest neighbor cell order between a target cell and its neighbor belonging to a different clonal population respectively. *In vivo* image analysis was carried out at pixel-level, in contrast to the cell-level in *in vitro* quantification. In this case, Dispersal Score refers to the least Euclidean distance between a target pixel and its neighbor belonging to a different clonal population because establishing exact nuclei positions in tissue sections was challenging. The code developed for this analysis is based on our previous work ^31^ and is available at https://github.com/SheratonMV/dispersion .

#### Data analysis

Cohort and sample overviews and visualizations were implemented in the R2 Genomics Analysis and Visualization Platform (https://r2.amc.nl), together with sample annotations.

To associate gene expression with experimentally obtained Dispersal Scores of CRC lines, we used a combined dataset ^83^ comprising cell line RNAseq datasets (GSE36133,^72^ GSE100478,^73^^,^^74^ GSE59857,^75^^,^^76^ GSE68950,^84^ GSE100549,^73^ and GSE100479 ^73^). To examine differences between normal and tumor tissues, we used the GSE199057 dataset.^47^ A microarray meta-dataset ^48^ containing GSE4183,^49^ GSE8671,^50^ GSE9348,^51^ GSE15960,^52^ GSE20916,^53^ GSE21510,^54^ GSE22598,^55^ GSE23194,^77^ GSE23878,^56^ GSE32323,^57^ GSE33113,^58^ and GSE37364 ^59^ was used to correlate the dispersal signature with adenoma and cancer samples. To assess dispersal signature expression across various cancers, we used datasets GSE62452,^78^ GSE4290,^79^ GSE3189,^80^ GSE53757.^81^ To evaluate whether the dispersal signature differs between non-relapse and relapse tissue samples, we utilized the GSE14333 dataset.^61^ The Cancer Genome Atlas (TCGA) Colon-Rectum Adenocarcinoma (COAD-READ) dataset ^60^ was used to correlate dispersal with overall and recurrence-free survival probabilities. Relapse-free survival probabilities were investigated using the GSE14333 ^61^ and GSE39582 ^82^ datasets.

### Quantification and statistical analysis

Statistical analyses were performed using GraphPad Prism 10.2.0. Significance was assessed using unpaired Student's t-tests for comparisons between two groups (Figures 1D, 1E, 2D, 2E, 3A–3F, 4B–4E, 4G, 4H, S3E, and S4A–S4D), ANOVA followed by a post-hoc test for multiple group comparisons (Figures 3F, 4A, 4C, 4F, S4E, S4F, S4I, and S4M), and the chi-square test for survival analysis (Figures 4I–4K, S4G, S4H, and S4J–S4L). "Ns", not significant, "∗", p<0.05, "∗∗", p<0.01, "∗∗∗", p<0.001, and "∗∗∗∗", p<0.0001. All *in vitro* and *in vivo* experiments were conducted with three biological replicates (n= 3), unless stated in the figure legends.

## Supplemental information

Document S1. Figures S1–S4 and Methods S1

Table S1. Genes correlated with *in vitro* and *in vivo* dispersal scores, related to Figure 3 Excel file containing additional data too large to fit in a PDF.

Table S2. EMT genes, related to Figure S4 Excel file containing additional data too large to fit in a PDF.
