# Supplementary material for: Clonal dispersal is associated with tumor heterogeneity and poor prognosis in colorectal cancer
Source: iScience. 2025 Apr 10;28(5):112403. doi: 10.1016/j.isci.2025.112403 (PMC12051713; doi:10.1016/j.isci.2025.112403)
Supplement: Document S1. Figures S1–S4 and Methods S1 [file mmc1.pdf]

iScience, Volume *28*

Supplemental information

Clonal dispersal is associated with tumor heterogeneity and poor prognosis in colorectal cancer

Selami Baglamis, Vivek M. Sheraton, Sanne M. van Neerven, Adrian Logiantara, Lisanne E. Nijman, Laura A. Hageman, Nicolas Léveillé, Clara C. Elbers, Maarten F. Bijlsma, Louis Vermeulen, Przemek M. Krawczyk, and Kristiaan J. Lenos

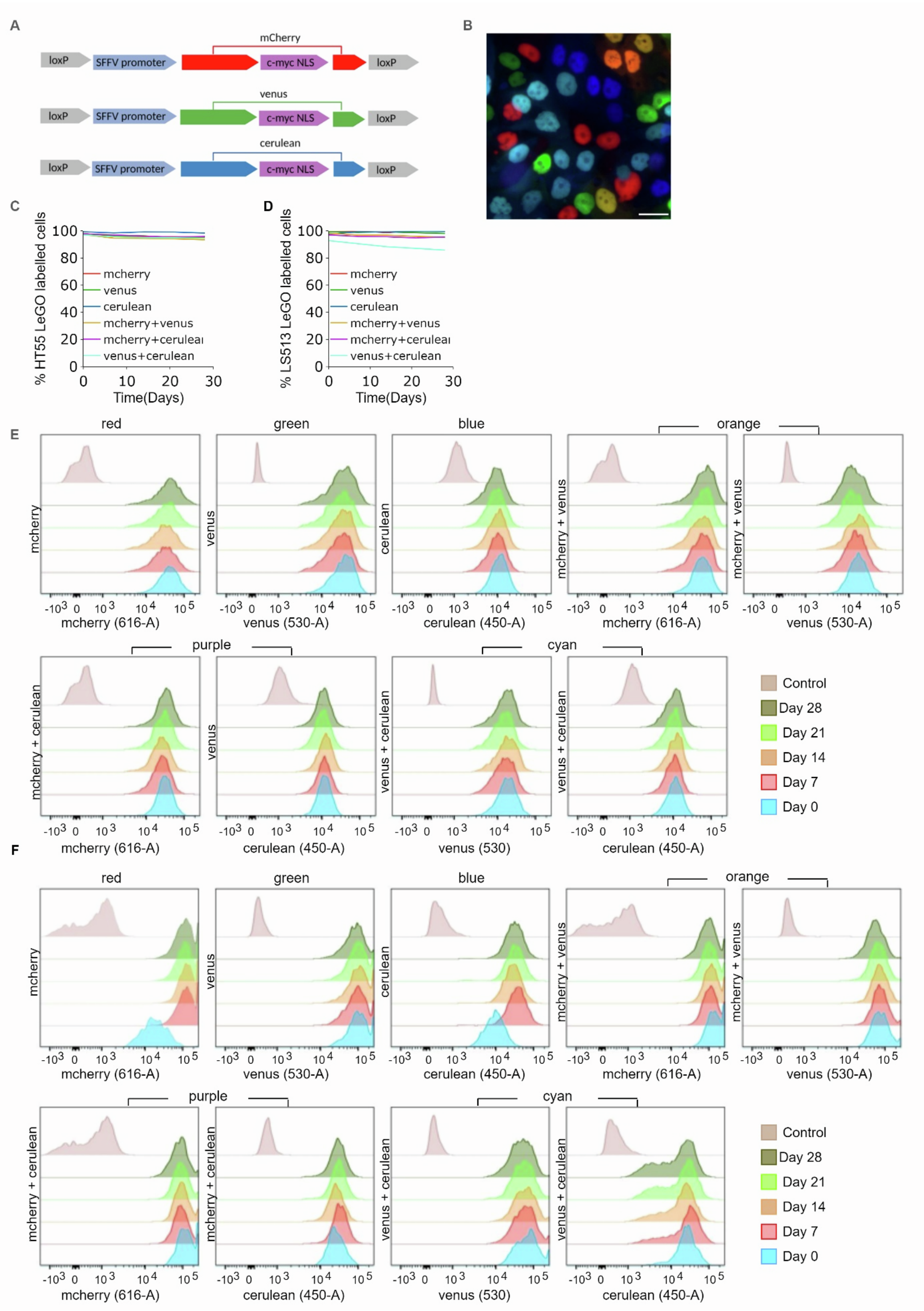
A
mCherry
loxP
SFFV promoter
c-myc NLS
loxP
venus
cerulean
B
C
% HT55 LeGO labelled cells
100
80
60
40
20
0
mcherry
venus
cerulean
mcherry+venus
mcherry+ceruleaı
venus+cerulean
0 10 20 30
Time(Days)
D
% LS513 LeGO labelled cells
Time(Days)
E
red
green
blue
orange
mcherry
venus
cerulean
mcherry + venus
mcherry (616-A)
venus (530-A)
cerulean (450-A)
purple
cyan
mcherry + cerulean
venus + cerulean
venus (530)
Control
Day 28
Day 21
Day 14
Day 7
Day 0
F
red
green
blue
orange
purple
cyan
mcherry + cerulean
venus + cerulean
Control
Day 28
Day 21
Day 14
Day 7
Day 0

Supplementary Figure 1. Expression of LeGO-NLS in transduced cells is stable over time. *(**A**) Linear representation of LeGO-NLS constructs, c-MYC NLS sequence cloned into plasmids [S1-4]. (**B**) Representative image of LeGO-NLS labeled CAR1 cells; scale bar: 20 μm. (**C, D, E, F**) Stability of LeGO-NLS fluorescent tags over time assessed by FACS. Day 0 refers to the initial FACS assessment conducted after a few passages following LeGO-NLS cell sorting. (**C, D**) Changes in the percentage of HT55 and LS513 labeled subpopulations. (**E, F**) The raw intensity of HT55 and LS513 labeled subpopulations over time, respectively.*

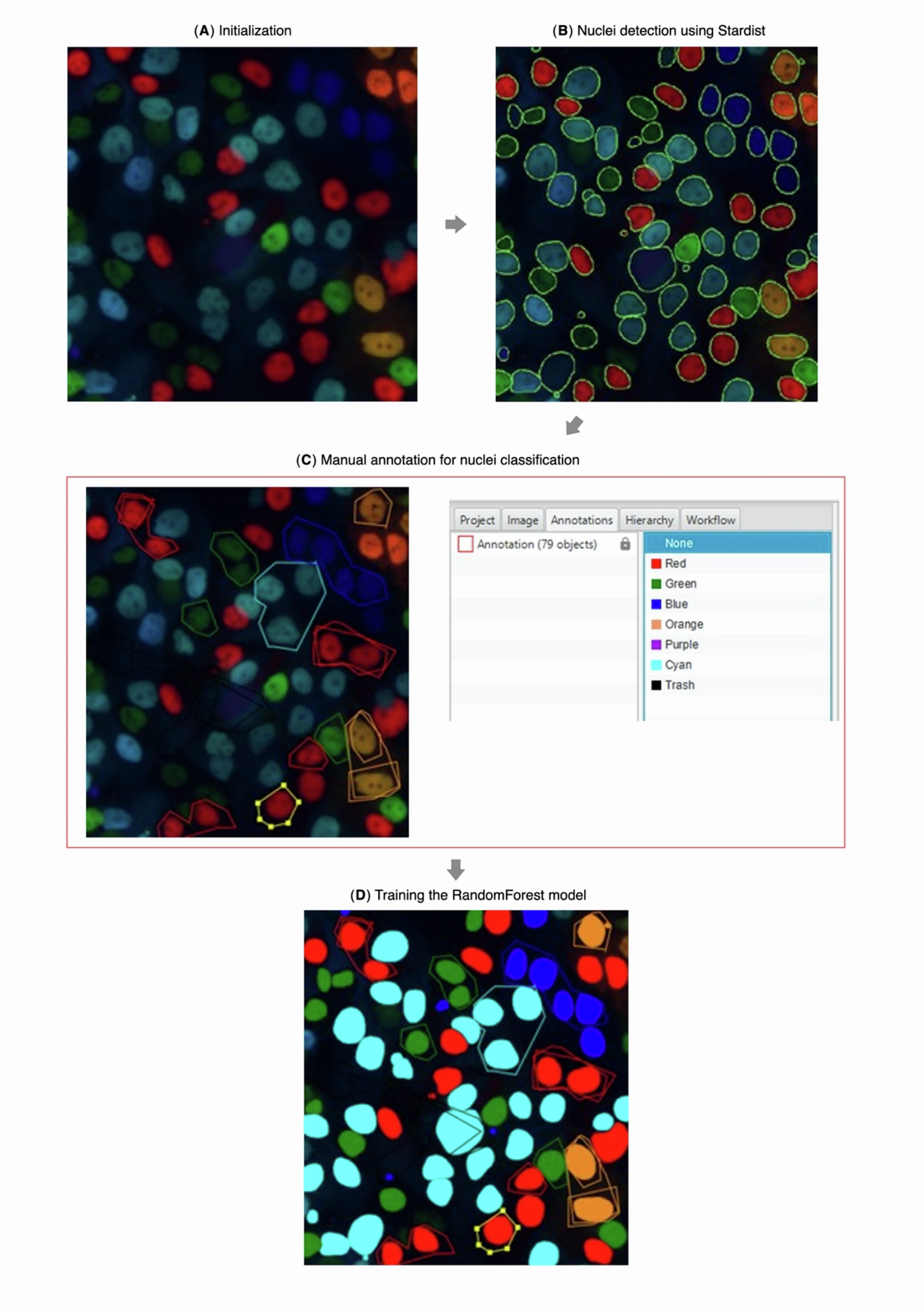
(A) Initialization
(B) Nuclei detection using Stardist
(C) Manual annotation for nuclei classification
Project
Image
Annotations
Hierarchy
Workflow
Annotation (79 objects)
None
Red
Green
Blue
Orange
Purple
Cyan
Trash
(D) Training the RandomForest model

Supplementary Figure 2. Qupath image analysis procedure. *The image analysis was conducted using the open-source software QuPath, available at https://qupath.github.io/. (**A**) Initialization: create a new project in QuPath and load the images into an empty directory. (**B**) Nuclei Detection using Stardist: Employ QuPath's built-in deep learning algorithm, Stardist, to select the well area on the plate and detect nuclei. (**C**) Manual Annotation for Nuclei Classification: Manually annotate different nuclei in QuPath by creating objects and grouping the detected nuclei into different color-coded categories. Any incorrectly detected elements, such as dirt or debris, should be assigned to a 'trash' group. (**D**)Training the RandomForest Model: Use the annotated color groups to train RandomForest and apply it to all images.*

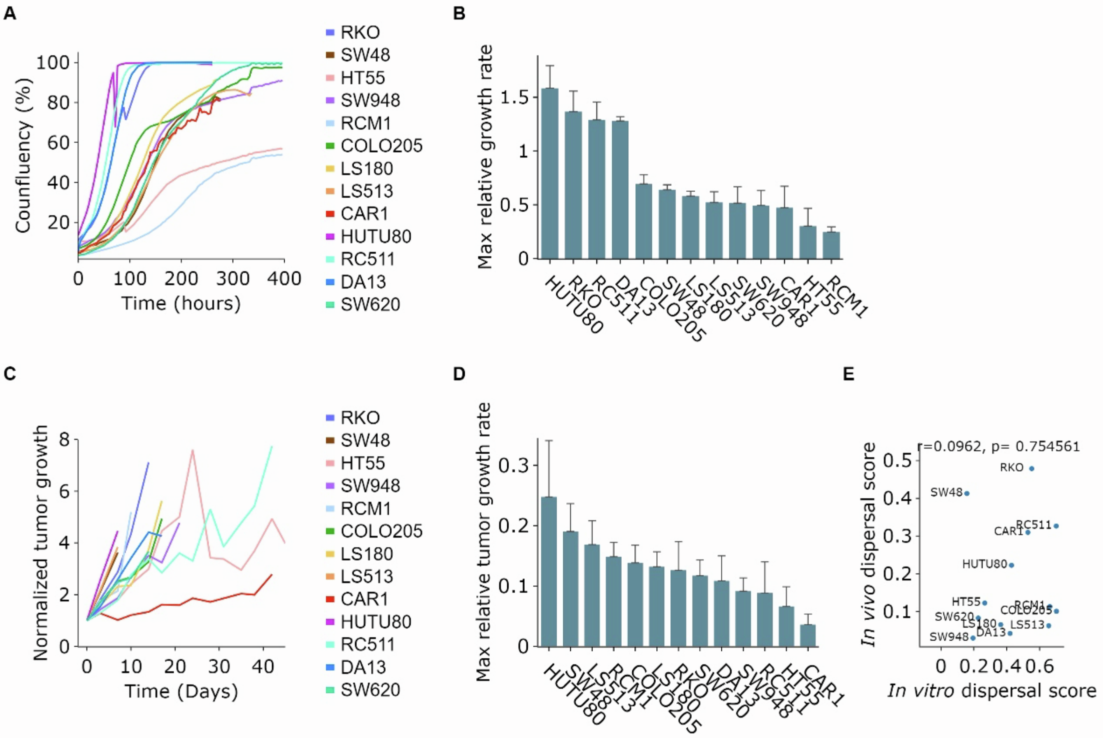
A
B
C
D
E
RKO
SW48
HT55
SW948
RCM1
COLO205
LS180
LS513
CAR1
HUTU80
RC511
DA13
SW620
Counfluency (%)
Time (hours)
Max relative growth rate
Normalized tumor growth
Time (Days)
Max relative tumor growth rate
r=0.0962, p= 0.754561
In vivo dispersal score
In vitro dispersal score

Supplementary Figure 3. Impact of cell proliferation and tumor growth rates on Dispersal Score. *(**A**) Proliferation rates of LeGO-NLS cell lines. Cells were monitored and analyzed using IncuCyte S3 automatic cell imager at 4-hour intervals. (**B**) Maximum relative growth rate for each cell line, determined by fitting a linear function to the exponential part of the growth curve from (**A**) and calculating its slope. (**C**) Normalized tumor growth over time. Day 0 is the first time when tumors were measurable. (**D**) The maximum relative growth rate for each tumor, determined from (**C**). The growth rate was calculated by using the exponential phase of each tumor individually and then averaging these values for each tumor type. (**E**) Pearson's correlation between the Dispersal Scores obtained from in vitro and in vivo quantification.*

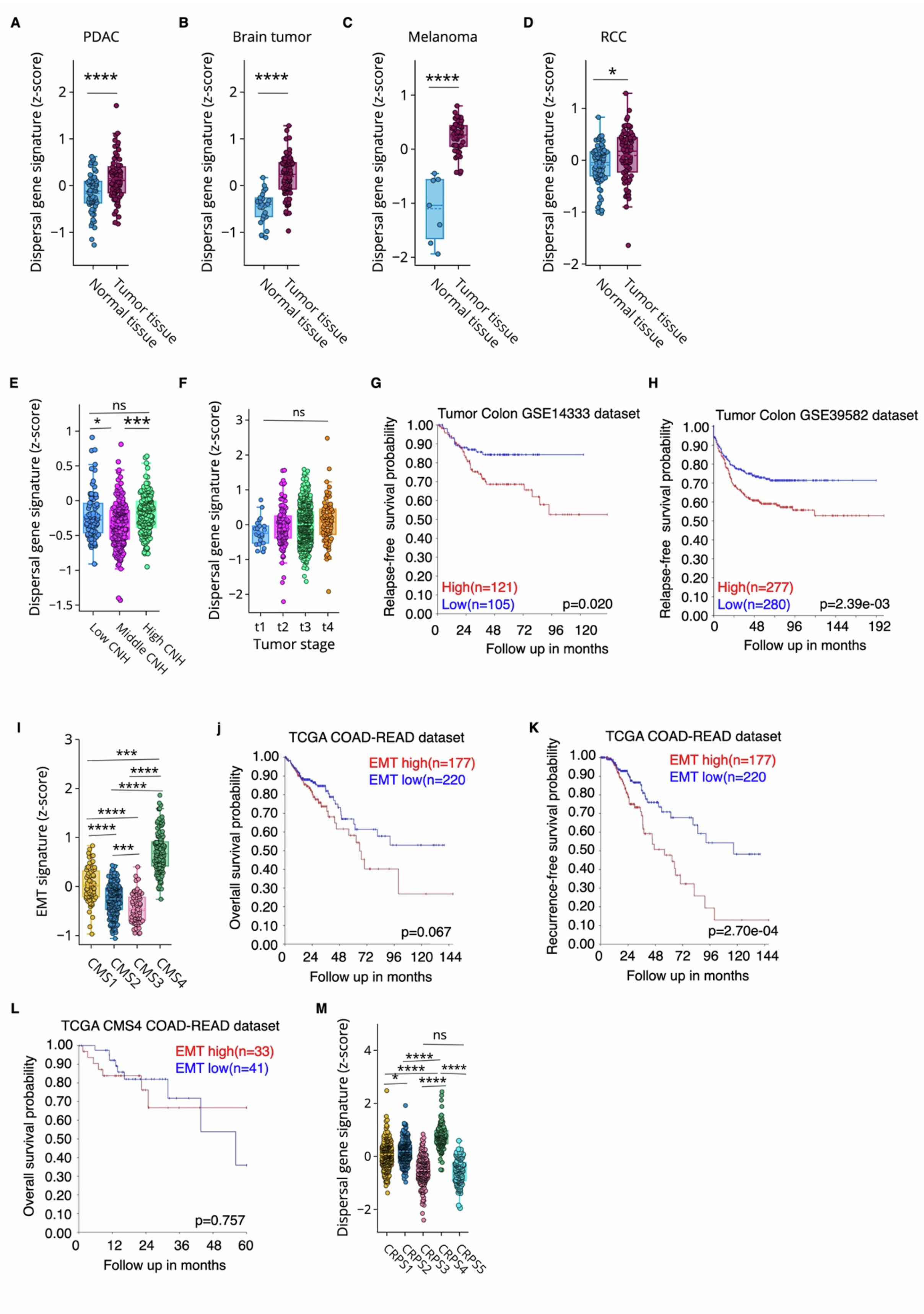

A
PDAC
B
Brain tumor
C
Melanoma
D
RCC
Dispersal gene signature (z-score)
****
*
Normal tissue
Tumor tissue
E
F
G
H
ns
***
Low CNH
Middle CNH
High CNH
t1
t2
t3
t4
Tumor stage
Tumor Colon GSE14333 dataset
Relapse-free survival probability
High(n=121)
Low(n=105)
p=0.020
Follow up in months
Tumor Colon GSE39582 dataset
High(n=277)
Low(n=280)
p=2.39e-03
I
EMT signature (z-score)
CMS1
CMS2
CMS3
CMS4
j
TCGA COAD-READ dataset
Overall survival probability
EMT high(n=177)
EMT low(n=220
p=0.067
K
TCGA COAD-READ dataset
Recurrence-free survival probability
EMT high(n=177)
EMT low(n=220
p=2.70e-04
L
TCGA CMS4 COAD-READ dataset
EMT high(n=33)
EMT low(n=41)
p=0.757
M
CRPS1
CRPS2
CRPS3
CRPS4
CRPS5

Supplementary Figure 4. Expression of dispersal signature correlates with clinical outcome** for (**A**) Pancreatic ductal adenocarcinoma (PDAC) in GSE62452 [S5], (**B**) Brain tumor in GSE4290 dataset [S6], (**C**) Melanoma in GSE3189 dataset [S7], (**D**) Renal cell carcinoma (RCC) in dataset* GSE53757 *[S8]. (**E**) Dispersal gene signature (z-score) in MSS CRCs, with either low, intermediate or high copy number heterogeneity (CNH) (TCGA COAD-READ dataset) [S9]. (**F**) The relation between tumor stage and dispersal gene signature (z-score) (TCGA COAD-READ dataset) [S10]. (**G, H**) Relapse-free survival probability of patients with either high or low dispersal gene signature in tumor colon GSE14333 [S11] and GSE39582 [S12] datasets, respectively. (**I**) EMT signature in CMS1 (n=68), CMS2 (n=207), CMS3 (n=64) and CMS4 (n=118) CRCs (TCGA COAD-READ dataset) [S13]. Each dot represents a different tumor sample. (**J**) Overall survival probability and (**K**), recurrence-free survival probability of CRC patients with either high or low EMT signature (TCGA COAD-READ dataset) [S13]. (**L**) Overall survival probability of CMS4 patients with either high or low EMT signature (TCGA COAD-READ dataset) [S13]. (**M**) Dispersal gene signature in CRPS1 (n=301), CRPS2 (n=294), CRPS3 (n=221), CRPS4 (n=130) and CRPS5 (114) subtypes [S14]. Each dot represents a different tumor sample. Significance was assessed using unpaired Student's t-tests for comparisons between two groups, ANOVA followed by a post-hoc test for multiple group comparisons, and the chi-square test for survival analysis. "Ns", not significant, "*", p<0.05, "**", p<0.01, "", p<0.001, and "****", p<0.0001.*

Supplementary Data / Method 1: Codes in Qupath

```
import qupath.ext.stardist.StarDist2D
def server = getCurrentServer()
import qupath.lib.gui.QuPathGUI
def projectpath = QuPathGUI.getInstance().getProject().getBaseDirectory()
// Specify the model file (you maybe need to change this!)
var pathModel = projectpath.toString()+'/models/dsb2018_heavy_augment.pb'
def cal = server.getPixelCalibration()

var downscale=1
var threshold=0.5 // Probability (detection) threshold
var cellexpansion=0 // Approximate cells based upon nucleus expansion (0=off)
try {
var stardist = StarDist2D.builder(pathModel)
.preprocess (
ImageOps.Channels.maximum()
)
.threshold(threshold) // Probability (detection) threshold
//.channels(0) // Select detection channel
.normalizePercentiles(1, 99) // Percentile normalization
.pixelSize(cal.getAveragedPixelSizeMicrons()*downscale) // Resolution for detection
.cellExpansion(cellexpansion) // Approximate cells based upon nucleus expansion
.cellConstrainScale(1.5) // Constrain cell expansion using nucleus size
.measureShape() // Add shape measurements
.measureIntensity() // Add cell measurements (in all compartments)
.includeProbability(true) // Add probability as a measurement (enables later filtering)
.doLog() // Use this to log a bit more information while running the script
//.tileSize(1024) // Specify width & height of the tile used for prediction
//.ignoreCellOverlaps(false) // Set to true if you don't care if cells expand into one another
//.nThreads(4) // Limit the number of threads used for (possibly parallel) processing
//.simplify(1) // Control how polygons are 'simplified' to remove unnecessary vertices
//.createAnnotations() // Generate annotation objects using StarDist, rather than detection
objects
//.constrainToParent(false) // Prevent nuclei/cells expanding beyond any parent annotations
(default is true)
.classify("Nuclei") // Automatically assign all created objects as 'Tumor'

.build()
// Run detection for the selected objects
clearSelectedObjects(true);
clearSelectedObjects();
resetSelection();
createAnnotationsFromPixelClassifier("Well", 10000.0, 0.0, "DELETE_EXISTING",
"SELECT_NEW")
var imageData = getCurrentImageData()
var pathObjects = getSelectedObjects()
if (pathObjects.isEmpty()) {
```

```
Dialogs.showErrorMessage("StarDist", "Please select a parent object!")
return
}
stardist.detectObjects(imageData, pathObjects)
setImageType('FLUORESCENCE');
runObjectClassifier("CAR1_test_rt_train");
println 'Done!'
}
catch(Exception e) {
Dialogs.showErrorMessage("Stardist",e.getMessage())
println 'Please Correct the Script!'
}
```

Supplementary references

1. Dang, C. V, and Lee, W.M. (1988). Identification of the human c-myc protein nuclear translocation signal. Mol. Cell. Biol. *8*, 4048–4054. DOI: 10.1128/mcb.8.10.4048-4054.1988
2. Qian, H., Baglamis, S., Redeker, F., Raaijman, J., Hoebe, R.A., Sheraton, V.M., Vermeulen, L., and Krawczyk, P.M. (2023). High-Content and High-Throughput Clonogenic Survival Assay Using Fluorescence Barcoding. Cancers (Basel) *15*, 4772. doi: 10.3390/cancers15194772
3. Baglamis, S., Saha, J., der Heijden, M. van, Miedema, D.M., van Gent, D., Krawczyk, P.M., Vermeulen, L., and Sheraton, V.M. (2023). A Novel High-Throughput Framework to Quantify Spatio-Temporal Tumor Clonal Dynamics. In https://doi.org/10.1007/978-3-031-36024-4_28.
4. Baglamis, S., Sheraton, V.M., Meijer, D., Qian, H., Hoebe, R.A., Lenos, K.J., Betjes, M.A., Betjes, M.A., Tans, S., van Zon, J., et al. (2023). Using picoliter droplet deposition to track clonal competition in adherent and organoid cancer cell cultures. Sci. Rep. *13*, 18832. doi: 10.1038/s41598-023-42849-w.
5. Yang, S., He, P., Wang, J., Schetter, A., Tang, W., Funamizu, N., Yanaga, K., Uwagawa, T., Satoskar, A.R., Gaedcke, J., et al. (2016). A Novel MIF Signaling Pathway Drives the Malignant Character of Pancreatic Cancer by Targeting NR3C2. Cancer Res. *76*. DOI: 10.1158/0008-5472.CAN-15-2841
6. Sun, L., Hui, A.-M., Su, Q., Vortmeyer Alexander and Kotliarov, Y., Pastorino, S., Passaniti, A., Menon, J., Walling, J., Bailey, R., Rosenblum, M., et al. (2006). Neuronal and glioma-derived stem cell factor induces angiogenesis within the brain. Cancer Cell *9*, 287–300. DOI: 10.1016/j.ccr.2006.03.003
7. Talantov, D., Mazumder, A., Yu, J.X., Briggs, T., Jiang, Y., Backus, J., Atkins, D., and Wang, Y. (2005). Novel genes associated with malignant melanoma but not benign melanocytic lesions. Clin. Cancer Res. *11*. DOI: 10.1158/1078-0432.CCR-05-0683
8. von Roemeling, C.A., Radisky, D.C., Marlow, L.A., Cooper, S.J., Grebe, S.K., Anastasiadis, P.Z., Tun, H.W., and Copland, J.A. (2014). Neuronal pentraxin 2 supports clear cell renal cell carcinoma by activating the AMPA-selective glutamate receptor-4. Cancer Res. *74*. DOI: 10.1158/0008-5472.CAN-14-0210
9. van Dijk, E., van den Bosch, T., Lenos, K.J., El Makrini, K., Nijman, L.E., van Essen, H.F.B., Lansu, N., Boekhout, M., Hageman Joris H and Fitzgerald, R.C., Punt, C.J.A., et al. (2021). Chromosomal copy number heterogeneity predicts survival rates across cancers. Nat. Commun. *12*, 1–12. doi: 10.1038/s41467-021-23384-6.
10. Liberzon, A., Birger, C., Thorvaldsdóttir Helga and Ghandi, M., Mesirov, J.P., and Tamayo, P. (2015). The Molecular Signatures Database (MSigDB) hallmark gene set collection. Cell Syst *1*, 417–425. DOI: 10.1016/j.cels.2015.12.004
11. Bressan, D., Battistoni, G., and Hannon, G.J. (2023). The dawn of spatial omics. Science (1979) *381*, eabq4964. DOI: 10.1126/science.abq4964
12. Medico, E., Russo, M., Picco, G., Cancelliere, C., Valtorta, E., Corti, G., Buscarino, M., Isella, C., Lamba, S., Martinoglio, B., et al. (2015). The molecular landscape of colorectal cancer cell lines unveils clinically actionable kinase targets. Nat. Commun. *6*. DOI: 10.1038/ncomms8002
13. Kirk, S., L.Y., S.C.A., L.S., R.C., B.E., & F.J. (2016). The Cancer Genome Atlas Colon Adenocarcinoma Collection (TCGA-COAD) (Version 3) [Data set]. https://doi.org/doi.org/10.7937/K9/TCIA.2016.HJJHBOXZ.
14. Nunes, L., Li, F., Wu, M., Luo, T., Hammarström, K., Torell, E., Ljuslinder, I., Mezheyeuski, A., Edqvist, P.H., Löfgren-Burström, A., et al. (2024). Prognostic genome and transcriptome

signatures in colorectal cancers. Nature 2024 633:8028 *633*, 137–146. https://doi.org/10.1038/s41586-024-07769-3.
